# Validation of the Family Caregiver Relationship Quality Scale in Long-Term Care Facilities in Taiwan

**DOI:** 10.3390/healthcare14081068

**Published:** 2026-04-17

**Authors:** Pai-Yueh Chen, Ying-Hua Chao, Yao-Ching Huang, Shi-Hao Huang, Ren-Jei Chung, Pi-Ching Yu, Bing-Long Wang, Hsiu-Ju Chang, Pi-Chen Chang, Shu-Min Huang, Chao-Hsi Huang

**Affiliations:** 1 School of Nursing, College of Nursing, Taipei Medical University, Taipei 11031, Taiwan; xuwuxiangchun92@gmail.com; 2 Department of Nursing, Yuanpei University of Medical Technology, Hsinchu 30015, Taiwan; chaoyh211@gmail.com; 3 School of Public Health, College of Public Health, National Defense Medical University, Taipei 11490, Taiwan; 4 Department of Chemical Engineering and Biotechnology, National Taipei University of Technology (Taipei Tech), Taipei 10608, Taiwan; 5 Graduate Institute of Medical Sciences, College of Medicine, National Defense Medical University, Taipei 11490, Taiwan; 6 Department of Health Administration, Asia University, Taichung 41354, Taiwan; 7 Department of Nursing, College of Nursing, National Yang Ming Chiao Tung University, Taipei 112304, Taiwan; 8 Department of Nursing, Efficient Smart Care Research Center, College of Nursing, National Yang Ming Chiao Tung University, Taipei 112304, Taiwan; 9 Department of Infection Control, Taipei Medical University Hospital, Taipei 11031, Taiwan; 10 Department of Computer Science and Information Engineering, National Ilan University, Yilan 26047, Taiwan

**Keywords:** long-term care, family caregiver, relationship quality, shared decision-making, scale validation, psychometrics, Taiwan

## Abstract

**Background:** Family caregivers remain closely involved in communication, care planning, and shared decision-making in long-term care (LTC) facilities. In this context, the quality of the relationship between family caregivers and professional staff may influence trust, collaboration, and satisfaction with care. However, few instruments have been specifically adapted to assess caregiver–staff relationship quality in Taiwanese LTC settings. **Objectives:** This study aimed to culturally adapt and preliminarily validate the Family Caregiver Relationship Quality (FCRQ) Scale for use in Taiwanese LTC facilities. **Methods:** A cross-sectional psychometric validation study was conducted with 205 primary family caregivers recruited from 20 LTC facilities in Taiwan. The original Relationship Quality Scale was adapted to the LTC context through contextual revision, expert review, bilingual verification, and pilot testing. Psychometric evaluation included confirmatory factor analysis, internal consistency assessment, convergent validity, and structural equation modelling with Bollen–Stine bootstrap correction to address potential non-normality. **Results:** The initial 16-item model required refinement, and three items with low standardized factor loadings were removed. The revised 13-item model met the prespecified fit criteria and showed acceptable internal consistency and convergent validity. The retained items reflected three conceptually related domains of relationship quality: trust, commitment, and satisfaction. Overall, the findings provided preliminary psychometric support for the adapted scale in Taiwanese LTC settings. **Conclusions:** The adapted FCRQ Scale may be a useful tool for assessing caregiver–staff relationship quality in Taiwanese long-term care facilities, particularly in the context of shared decision-making and family-centred care. Nevertheless, the findings should be interpreted as preliminary, and further validation in larger and more diverse samples is needed before broader clinical or research application.

## 1. Introduction

The rapid ageing of the global population has substantially increased the demand for long-term care (LTC) services, particularly for older adults living with chronic illness, disability, cognitive decline, or functional dependence who require sustained assistance in daily life and healthcare management [1,2,3]. In LTC settings, care is no longer limited to the resident alone; rather, it often involves ongoing interaction among residents, family caregivers, nurses, care staff, and other healthcare professionals. Even after institutional placement, family caregivers frequently remain deeply involved in care planning, communication, and treatment-related decision-making, while also experiencing considerable emotional, practical, and ethical burden [4,5]. In addition, unplanned hospitalizations and health deterioration are common among LTC residents and may further intensify caregiver stress, family uncertainty, and the need for timely decision-making [6,7,8].

Within this context, shared decision-making (SDM) has become an important component of patient- and family-centred care. SDM emphasizes collaboration between healthcare professionals and care recipients or their families to arrive at decisions that are not only clinically appropriate but also aligned with personal values, preferences, and care goals [9,10]. Although SDM has been widely discussed in acute care, outpatient medicine, and psychiatric settings, its implementation in LTC remains comparatively underdeveloped [11,12,13]. This gap is important because decision-making in LTC is often more relational, prolonged, and emotionally complex than in short-term medical encounters. However, many currently available SDM-related instruments were developed for acute or episodic healthcare settings and rely mainly on self-reports from patients or physicians, which may not adequately capture the sustained interpersonal dynamics between family caregivers and facility staff in LTC environments [14,15,16].

One key yet under-measured construct in LTC is the quality of the relationship between family caregivers and professional staff. In institutional care settings, the quality of this relationship may shape the degree of trust, communication, collaboration, and satisfaction involved in care planning and decision-making. A positive caregiver–staff relationship may facilitate mutual respect, reduce decisional conflict, and improve the consistency and acceptability of care decisions, whereas poor communication or low trust may contribute to dissatisfaction, emotional distress, and problematic care transitions [17,18,19]. For this reason, assessing relationship quality is important not only for understanding caregiver experience but also for improving SDM practices and family-centred care processes in LTC. Conceptually, relationship quality is often understood as a multidimensional construct that includes trust, commitment, and satisfaction, which together reflect the strength and stability of an ongoing collaborative relationship [20,21,22].

The concept of relationship quality was originally derived from the commitment–trust model in relationship marketing theory, which proposes that trust and commitment are central to the development and maintenance of enduring cooperative relationships [21]. This theoretical perspective was later extended into healthcare and psychosocial research to examine the quality of patient–provider and care-related relationships [23,24,25]. In Taiwan, Chung developed the original Relationship Quality (RQ) Scale within a shared decision-making framework to assess patients’ perceptions of trust, commitment, and satisfaction in relation to healthcare providers [26]. The original 16-item Chinese instrument demonstrated acceptable internal consistency and construct validity across three subscales, suggesting that it may provide a useful conceptual basis for measuring relational processes in healthcare [26].

Nevertheless, direct application of this instrument to LTC settings is not straightforward. Compared with hospital or outpatient contexts, LTC environments involve longer-term interaction, more frequent involvement of family members, and stronger influence from cultural expectations regarding family responsibility, reciprocity, and relational harmony. Therefore, a culturally and contextually adapted measure is needed to capture how family caregivers perceive their relationship with staff in Taiwanese LTC facilities. Although subsequent work has explored adaptation of the RQ framework in LTC contexts, the evidence base remains preliminary and further psychometric evaluation is needed to support its use in this setting [27].

Accordingly, the present study aimed to develop and validate a culturally adapted Family Caregiver Relationship Quality Scale for use in Taiwanese long-term care facilities. By adapting the original RQ framework to the LTC caregiving context and evaluating its psychometric properties, this study seeks to provide an initial evidence-based instrument for assessing caregiver–staff relationship quality in support of shared decision-making, family-centred care, and quality improvement in LTC systems.

## 2. Methods/Methodology

### 2.1. Study Design

This study employed a cross-sectional psychometric validation design to evaluate the reliability and validity of a culturally adapted Family Caregiver Relationship Quality Scale (FCRQ) in LTC facilities. The research process was guided by established recommendations for instrument development and validation in health sciences, which emphasize sequential procedures including construct definition, cultural and linguistic adaptation, expert assessment of content relevance and clarity, pilot testing, and psychometric evaluation of reliability and validity [20,24]. In the present study, these recommendations were implemented through adaptation of the original scale to the LTC context, review by an expert panel, pilot testing with family caregivers, and formal psychometric testing using CFA and related validity indicators. Specifically, the revised Methods section now explains not only the cited guidance itself, but also the correspondence between each recommended step and the procedures used in this study. The procedure included linguistic adaptation, expert review, pilot testing, and psychometric evaluation through CFA and SEM.

### 2.2. Setting and Participants

The study was conducted across 20 LTC facilities located in northern, central, and southern Taiwan between December 2023 and January 2024. Facilities were purposively selected to ensure diversity in ownership (public vs. private) and size (50–300 beds).

Participants were primary family caregivers of institutionalized residents who met the following inclusion criteria:

1. Aged 20 years or older.
2. Identified as the principal decision-maker or individual responsible for the resident’s daily or medical care.
3. Able to communicate in Mandarin Chinese.
4. Provided informed written consent.

Caregivers who were paid employees, had cognitive or communication difficulties, or withdrew participation were excluded.

Sample size estimation followed Kline’s recommendation for SEM, requiring at least 10 participants per estimated parameter and a minimum of 200 valid responses for stable model estimation and reasonable generalizability [25]. Accordingly, the final sample of 205 participants met the minimum recommended threshold for CFA/SEM. However, because larger samples are generally preferable when distributional assumptions may not be fully satisfied, the sample size in the present study should be interpreted as minimally adequate rather than optimal.

### 2.3. Instrument Development and Adaptation

The RQ Scale was originally developed by Chung (2018) under the supervision of Prof. Rong-Fang Chen at National Kaohsiung University of Applied Sciences [17]. The scale was grounded in the Trust–Commitment Theory [21] and the shared decision-making (SDM) conceptual framework [9]. It measures three constructs—trust (6 items), commitment (6 items), and satisfaction (4 items)—rated on a 5-point Likert scale ranging from 1 (strongly disagree) to 5 (strongly agree). The original instrument demonstrated acceptable psychometric properties, with an overall Cronbach’s alpha of 0.87 and subscale coefficients ranging from 0.72 to 0.81 [28].

For this study, written authorization was obtained from the original developer to adapt the RQ Scale for use in LTC settings. Following approval from the Taipei Medical University Joint Institutional Review Board (IRB No. N202307042), the adaptation process was conducted in accordance with established recommendations for instrument development and validation in health sciences, including contextual item revision, expert review, pilot testing, and subsequent psychometric evaluation [20,24]. Because the original scale was developed for healthcare encounters rather than institutional LTC settings, selected items were linguistically and contextually modified to improve applicability to Taiwanese LTC facilities while preserving the conceptual meaning of the original instrument.

The adaptation process included translation review, cultural adaptation, expert evaluation, and pilot testing. A five-member expert panel comprising three nursing professors and two senior LTC administrators reviewed the translated and adapted items after the forward–backward translation process. The panel did not serve as the primary translators; rather, their role was to evaluate semantic clarity, contextual appropriateness, and conceptual consistency with the original instrument, and to provide consensus-based recommendations for item refinement. To improve contextual relevance, terms such as “physician/medical team” were revised to “care staff in the facility,” and “treatment outcomes” were changed to “care outcomes.” The revised wording was subsequently reviewed by bilingual experts proficient in Mandarin Chinese and English to assess semantic clarity and conceptual equivalence between the original and adapted versions [29]. However, the exact number of bilingual experts was not formally documented, and a formal double-blind back-translation procedure was not performed. Instead, semantic and conceptual equivalence were strengthened through bilingual review and consensus-based expert evaluation.

A pilot test was then conducted with 30 family caregivers recruited from LTC facilities to assess item clarity, comprehensibility, and response consistency. Participants completed the preliminary version of the instrument and were invited to identify any wording that was unclear or difficult to interpret. Minor wording adjustments were made based on pilot feedback prior to formal psychometric testing. The pilot version demonstrated good preliminary internal consistency, with a Cronbach’s alpha of 0.91. In the present study, these three related domains were retained as the conceptual basis of the adapted instrument.

### 2.4. Data Collection Procedures

Data collection occurred during scheduled family visits under facility permission. Trained research assistants explained the study’s purpose, obtained informed consent, and distributed structured questionnaires. Each participant completed the survey independently within approximately 15–20 min.

Participation was voluntary and anonymous. No incentives were provided. Data confidentiality was maintained using coded identifiers. Ethical approval was obtained from the Taipei Medical University Joint IRB (No. N202307042) in accordance with the Declaration of Helsinki (2013 revision).

### 2.5. Statistical Analysis

All analyses were performed using SPSS version 26.0 (IBM Corp., Armond, NY, USA) and AMOS version 24.0, Smart PLS version 4.0.9.2 (Smart PLS GmbH, Bönningstedt, Germany). Descriptive statistics (mean, SD, percentage) summarized participant characteristics. Psychometric validation followed established recommendations for scale evaluation [20,23,24,30,31]:

- Internal consistency was evaluated using Cronbach’s alpha and composite reliability. In general, values in the range of approximately 0.80–0.90 are often considered indicative of good internal consistency, although interpretation should take scale purpose and item characteristics into account [32,33].
- Composite reliability (CR) and average variance extracted (AVE): CR ≥ 0.70 and AVE ≥ 0.50 indicated good convergent validity [33].
- CFA: evaluated using standardized factor loadings and multiple model-fit indices. Standardized loadings of at least 0.60 were considered acceptable. For model fit, values approaching or exceeding 0.95 for CFI, TLI, and GFI, together with SRMR and RMSEA values below 0.08, were considered supportive of adequate model fit [34,35].
- Discriminant validity: assessed using the Fornell–Larcker criterion, where AVE for each construct exceeded the squared correlations between constructs [33].
- SEM: was conducted using Maximum Likelihood Estimation. Because CFA model estimation may be affected when multivariate normality assumptions are not fully met, Bollen–Stine bootstrapping was additionally applied to evaluate model fit under potential non-normal data conditions [36,37,38].

If the initial CFA indicated inadequate item performance or model fit, item retention was evaluated primarily on the basis of standardized factor loadings, conceptual relevance, and overall model interpretability. Final model evaluation was then conducted after refinement using the same prespecified psychometric criteria.

## 3. Results

### 3.1. Participant Characteristics

A total of 205 primary caregivers participated in this study, including 112 residing in Taipei City and 93 residing outside Taipei City. The sociodemographic characteristics of the overall sample and the two residence-based subgroups are presented in Table 1.

### 3.2. Reliability and Validity of the Relationship Quality (RQ) Scale

CFA was first conducted on the original 16-item model using AMOS 24 with Maximum Likelihood Estimation and Bollen–Stine bootstrap correction to account for potential non-normality in the data [39,40,41]. The initial 16-item model demonstrated suboptimal fit according to the prespecified criteria, indicating that model refinement was necessary. Examination of item performance showed that three items—RQ9 and RQ10 from the commitment subscale and RQ14 from the satisfaction subscale—had standardized factor loadings below the prespecified cutoff of 0.60 and were therefore removed.

After removal of these three items, the refined 13-item model met the recommended fit criteria. Standardized factor loadings ranged from 0.63 to 0.81, composite reliability (CR) was 0.934, and average variance extracted (AVE) was 0.523, exceeding the recommended thresholds for convergent validity [42]. Internal consistency for the overall revised RQ scale was acceptable (Cronbach’s *α* = 0.84). Notably, only one of the four original satisfaction items was removed, and the remaining three satisfaction items (RQ13, RQ15, and RQ16) were retained in the final model. Detailed standardized factor loadings and summary psychometric estimates for the revised scale are presented in Table 2.

### 3.3. Model Fit Indices

The CFA indicated that the revised 13-item model met the recommended fit criteria across multiple indices (Table 3) [43]. Overall, these findings supported the adequacy of the final measurement model and provided preliminary support for the construct validity of the adapted RQ Scale.

### 3.4. Confirmatory Factor Structure

**Figure 1:**
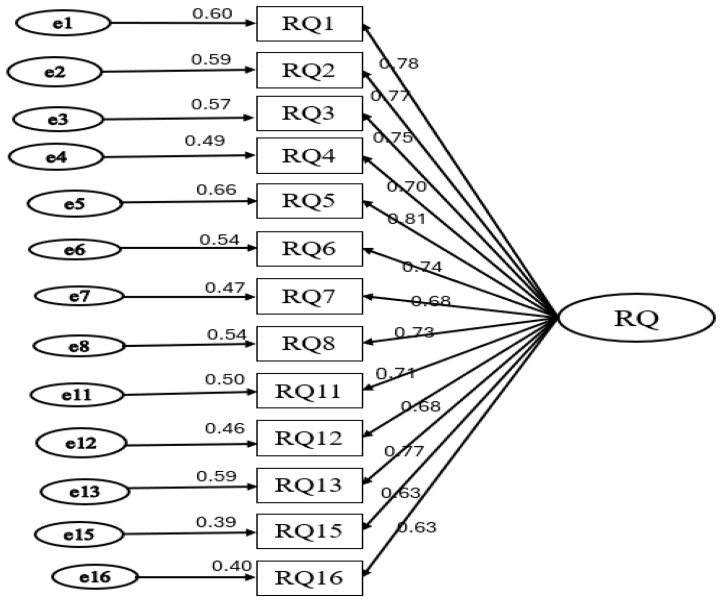
Confirmatory factor analysis of the revised Relationship Quality (RQ) Scale.

The initial 16-item model showed suboptimal fit according to the prespecified criteria, indicating that model refinement was required before the final model was established. Figure 1 presents the CFA model of the revised RQ Scale. After removal of three items with low standardized factor loadings (RQ9, RQ10, and RQ14), the refined 13-item model met the recommended fit criteria. Standardized factor loadings for the retained items ranged from 0.63 to 0.81, supporting the adequacy of the final measurement model in the present sample.

The figure shows the final CFA model for the revised 13-item RQ Scale. Standardized factor loadings ranged from 0.63 to 0.81, and all paths were statistically significant (*p* < 0.001). The refined model met the prespecified model-fit criteria, supporting the adequacy of the final measurement structure in this sample.

### 3.5. Summary of Results

Overall, the revised 13-item RQ model demonstrated acceptable internal consistency, satisfactory convergent validity, and fit indices that met the recommended criteria. After model refinement and Bollen–Stine bootstrap correction, the final measurement model remained stable in the present sample. Taken together, these findings provide preliminary psychometric support for the adapted RQ Scale as a measure of caregiver–staff relational quality in Taiwanese long-term care facilities.

## 4. Discussion

This study aimed to validate a culturally adapted FCRQ Scale for assessing the quality of caregiver–staff interactions in Taiwanese LTC facilities. The findings provide preliminary psychometric support for the culturally adapted 13-item version of the scale and suggest that the instrument may be useful for assessing caregiver–staff relationship quality in Taiwanese LTC settings.

### 4.1. Principal Findings

The confirmatory factor analysis indicated that the revised 13-item model met the recommended fit criteria in the present sample, supporting the adequacy of the final measurement model in the Taiwanese LTC context. The standardized factor loadings (0.63–0.81) and composite reliability (CR = 0.93) provided preliminary support for the internal coherence of the retained items, which reflected key relational attributes such as trust, commitment, and satisfaction that have previously been identified in healthcare relationship research [21,44]. These findings also provide preliminary support for the view that relationship quality is relevant to shared decision-making and sustained caregiver–staff partnerships in LTC settings [8,45,46].

The removal of RQ9 and RQ10 from the commitment domain and RQ14 from the satisfaction domain may reflect contextual differences between the original healthcare setting and institutional LTC environments. In LTC facilities, caregiver–staff relationships are often shaped by long-term interaction, continuity of care, family expectations, and culturally influenced perceptions of trust and reciprocity. Under these conditions, some items derived from acute or general healthcare encounters may be less salient or may not function in the same way when applied to sustained caregiving relationships. Although removal of RQ14 reduced the number of items in the satisfaction domain from four to three, the construct remained represented in the final model by three retained items, suggesting that this domain was still conceptually preserved. Although removal of RQ9, RQ10, and RQ14 improved model fit, it may also have narrowed the conceptual breadth of the original domains to some extent. This trade-off between statistical refinement and content coverage should be considered in future validation studies.

The findings also suggest that the originally proposed structure was not fully supported in its initial form in this sample. Rather than being interpreted as a failure of the underlying concept, this may indicate that the expression of relationship quality in Taiwanese LTC settings differs somewhat from that in the original development context. Because the present study was designed as an initial confirmatory validation of an adapted instrument, model refinement was undertaken within the CFA framework using prespecified psychometric criteria. Nevertheless, exploratory factor analysis may be informative in future studies with larger and more diverse samples to further examine the dimensionality of the adapted scale and to determine whether alternative structural solutions better represent caregiver–staff relationship quality in LTC settings [8,45,46].

### 4.2. Cultural Context and Theoretical Implications

The findings underscore the importance of cultural adaptation when applying psychosocial measurement tools in non-Western societies. In Taiwan’s collectivist culture, the caregiving dynamic is heavily influenced by filial piety, family obligation, and social harmony, which differ markedly from Western notions of individual autonomy [47,48,49].

Caregivers in East Asian LTC contexts often perceive relationship quality through moral reciprocity and trustworthiness rather than transactional satisfaction alone [50].

Hence, the translated and semantically adjusted items—particularly replacing “medical team” with “care staff”—align more closely with caregivers’ lived experiences and moral expectations in institutional care.

The validated model further reinforces Morgan and Hunt’s commitment–trust theory [51] in a healthcare framework, suggesting that sustained cooperation and perceived relational integrity are critical determinants of caregiver satisfaction. The integration of SDM theory within this model reflects a paradigm shift in nursing practice, from task-oriented care toward relational, value-based collaboration.

### 4.3. Practical Implications for Nursing and Policy

From a practical perspective, regular assessment of relationship quality may help nursing administrators and LTC staff identify communication difficulties or trust deficits between family caregivers and care staff, which are important factors influencing family engagement and shared decision-making in long-term care settings [52]. In addition, the use of a structured instrument may support the development of targeted interventions, such as communication training, family meetings, and relationship-centred care strategies, to improve collaboration between caregivers and facility staff [6]. At the policy level, relationship quality may also serve as a useful quality indicator for family-centred care and shared decision-making in LTC facilities, thereby contributing to quality improvement initiatives and person- and family-oriented service planning [53].

### 4.4. Comparison with Previous Research

Previous work by Chung (2018) conceptualized RQ in medical settings, identifying trust, commitment, and satisfaction as primary relational determinants [17,54]. Our study extends this framework by confirming that the same triadic structure applies in LTC institutions, albeit with culturally nuanced meanings. The RQ scale’s reliability (*α* = 0.84) and convergent validity (AVE = 0.52) are comparable or superior to international tools such as the Health Care Relationship Trust Scale (HCRTS) and Nurse–Patient Relationship Quality Index (NPRQI) [55,56]. These findings collectively affirm that relationship quality transcends healthcare settings yet must be contextually calibrated for cultural and structural differences.

### 4.5. Methodological Considerations

This study’s methodological strengths include a large sample size (*n* = 205), multi-site sampling, and the application of Bollen–Stine bootstrapping, which enhances the robustness of SEM results under non-normal data [39]. The use of content validation by experts, pilot testing, and confirmatory factor analysis aligns with international psychometric standards [20,31].

However, the study’s cross-sectional design limits causal inference regarding the relationship between RQ and downstream outcomes (e.g., caregiver stress, resident satisfaction). Future studies may adopt longitudinal or multi-level designs to explore causal pathways and institutional effects. Additionally, inclusion of care recipients’ perspectives and qualitative triangulation would enrich understanding of relational dynamics in LTC.

### 4.6. Limitations and Future Directions

Several limitations should be considered when interpreting the findings of this study. First, although participants were recruited from multiple long-term care facilities, the sample was limited to Mandarin-speaking family caregivers in Taiwan, which may restrict the generalizability of the findings to other linguistic, cultural, or healthcare-system contexts. Second, all responses were obtained through caregiver self-report, the findings may have been influenced by subjective interpretation, social desirability, or mood-related response tendencies, which could have affected perceived relationship quality ratings Third, the cross-sectional design precludes any inference regarding temporal stability or predictive relationships between relationship quality and downstream care outcomes.

Importantly, the present study should be interpreted as an initial psychometric validation of the adapted instrument rather than definitive evidence that the scale is fully established for broad clinical or research use. Although the findings provide preliminary support for the revised measurement model in this sample, a single study is insufficient to establish the instrument as psychometrically complete. Further replication is needed in larger and more diverse samples, including different regions, care settings, and caregiver populations. Additional validation procedures, such as test–retest reliability, external criterion validity, predictive validity, and measurement invariance testing across relevant subgroups, would further strengthen the evidence base for the adapted scale. Future research may also benefit from incorporating alternative data sources, such as observational assessments or staff-reported perspectives, to complement caregiver self-report and provide a more comprehensive understanding of caregiver–staff relationship quality in LTC settings.

## 5. Conclusions

The present study provides preliminary support for the use of the adapted RQ Scale in Taiwanese long-term care facilities. The revised instrument may offer a useful approach for examining caregiver–staff relationship quality within the framework of shared decision-making and family-centred care. Nevertheless, because this study represents an initial psychometric evaluation, further replication and additional validation are required to confirm its broader applicability across settings and populations.

## Figures and Tables

**Table 1:** Sociodemographic characteristics of primary caregivers by place of residence.

Variable	Taipei City (*n* = 112)	Outside Taipei City (*n* = 93)	Total (N = 205)
**Gender**			
Men	55 (49.1)	36 (38.7)	91 (44.4)
Women	57 (50.9)	57 (61.3)	114 (55.6)
**Age group (years)**			
20–29	16 (14.3)	19 (20.4)	35 (17.1)
30–39	13 (11.6)	12 (12.9)	25 (12.2)
40–49	17 (15.2)	10 (10.8)	27 (13.2)
50–59	16 (14.3)	14 (15.1)	30 (14.6)
60–69	21 (18.8)	14 (15.1)	35 (17.1)
70–79	12 (10.7)	15 (16.1)	27 (13.2)
80–89	17 (15.2)	9 (9.7)	26 (12.7)
**Residents’ identity**			
Low income	19 (17.0)	20 (21.5)	39 (19.0)
Medium to low income	5 (4.5)	0 (0.0)	5 (2.4)
Emergency resettlement	56 (50.0)	48 (51.6)	104 (50.7)
Others	32 (28.6)	25 (26.9)	57 (27.8)
**Education level**			
High school or below	54 (48.2)	52 (56.0)	106 (51.7)
College/University	57 (50.9)	34 (36.6)	91 (44.4)
Graduate school	1 (0.9)	7 (7.5)	8 (3.9)

**Note:** Percentages may not total 100% due to rounding.

**Table 2:** Standardized factor loadings and psychometric summary of the revised Relationship Quality scale.

Construct/Item	Standardized Factor Loading
**Trust**	
RQ1 The caregivers at this LTCF demonstrate reliability in their daily care duties.	0.78
RQ2 LTCFs for seniors have earned my confidence.	0.77
RQ3 I find the caregivers at this facility sincere in their interactions and care relationships.	0.75
RQ4 The staff at this LTCF exhibit strong moral character.	0.70
RQ5 Caregivers maintain high standards in their work performance.	0.81
RQ6 Care providers adhere to strong ethical principles in caregiving.	0.74
**Commitment**	
RQ7 I am dedicated to maintaining my commitment to this LTCF.	0.68
RQ8 This LTCF has made a lasting, positive impact on me.	0.73
RQ11 Ending my relationship with this LTCF would be difficult.	0.71
RQ12 I consciously chose this LTCF as a trustworthy option.	0.68
**Satisfaction**	
RQ13 The everyday care provided meets my standards.	0.77
RQ15 Overall, I am pleased with the care provided.	0.63
RQ16 My family’s experience exceeded expectations.	0.63
**Cronbach’s α**	0.84
**Composite reliability (CR)**	0.93
**Average variance extracted (AVE)**	0.52

**Note:** Standardized estimates are reported. CR ≥ 0.70 and AVE ≥ 0.50 indicate acceptable convergent validity.

**Table 3:** Model-fit indices of confirmatory factor analysis (CFA).

Fit Index	Ideal Criterion	Model Result
Bollen–Stine χ^2^	Smaller values indicate better fit	96.56
df	-	65
χ^2^/df	1–3	1.49
GFI	≥0.95	0.95
AGFI	≥0.90	0.94
RMSEA	<0.08	0.05
SRMR	<0.08	0.07
TLI (NNFI)	≥0.95	0.98
CFI	≥0.95	0.98

**Note:** Model fit was evaluated using multiple indices; values approaching or exceeding the recommended criteria were considered supportive of adequate model fit.

## Data Availability

The datasets generated and/or analyzed during the current study are available from the corresponding author upon reasonable request.
